# Involvement of opsins in mammalian sperm thermotaxis

**DOI:** 10.1038/srep16146

**Published:** 2015-11-05

**Authors:** Serafín Pérez-Cerezales, Sergii Boryshpolets, Oshri Afanzar, Alexander Brandis, Reinat Nevo, Vladimir Kiss, Michael Eisenbach

**Affiliations:** 1 Department of Biological Chemistry, The Weizmann Institute of Science, 7610001 Rehovot, Israel.; 2 Department of Biological Services, The Weizmann Institute of Science, 7610001 Rehovot, Israel

## Abstract

A unique characteristic of mammalian sperm thermotaxis is extreme temperature sensitivity, manifested by the capacity of spermatozoa to respond to temperature changes of <0.0006 °C as they swim their body-length distance. The identity of the sensing system that confers this exceptional sensitivity on spermatozoa is not known. Here we show that the temperature-sensing system of mammalian spermatozoa involves opsins, known to be G-protein-coupled receptors that act as photosensors in vision. We demonstrate by molecular, immunological, and functional approaches that opsins are present in human and mouse spermatozoa at specific sites, which depend on the species and the opsin type, and that they are involved in sperm thermotaxis via two signalling pathways—the phospholipase C and the cyclic-nucleotide pathways. Our results suggest that, depending on the context and the tissue, mammalian opsins act not only as photosensors but also as thermosensors.

In eukaryotes, temperature is sensed by temperature-sensitive ion channels, primarily belonging to the transient receptor potential (TRP) family. In addition, rhodopsin (Opsin-2) has been suggested as a possible thermosensor in Drosophila (refs 1,2 for reviews). A particularly interesting case of temperature sensing is thermotaxis of mammalian spermatozoa. In this process spermatozoa swim to a warmer temperature by actively changing their swimming direction according to the temperature gradient^3^,^4^,^5^,^6^. They are able to respond to extraordinarily small temperature differences. Thus, a human spermatozoon can respond to a temperature difference as small as <0.0006 °C when it swims its body-length distance^7^. This response was observed over a wide temperature range, at least 29–41 °C^7^. Consistent with the finding that temperature-sensitive ion channels shown to exist in mammalian spermatozoa (TRPM8 and heat-gated TRPV channels) are not involved in human sperm thermotaxis^8^, this extreme temperature sensitivity suggests that the thermosensors for mammalian sperm thermotaxis are not temperature-sensitive ion channels. After all, it is difficult to envisage how an ion channel can have such high temperature sensitivity over such a wide temperature range. If so, how is the temperature sensed? What is the identity of the sensing system that confers extreme temperature sensitivity on spermatozoa? Here we address these questions.

## Results

### GPCRs are involved in thermotaxis

**Figure 1:**
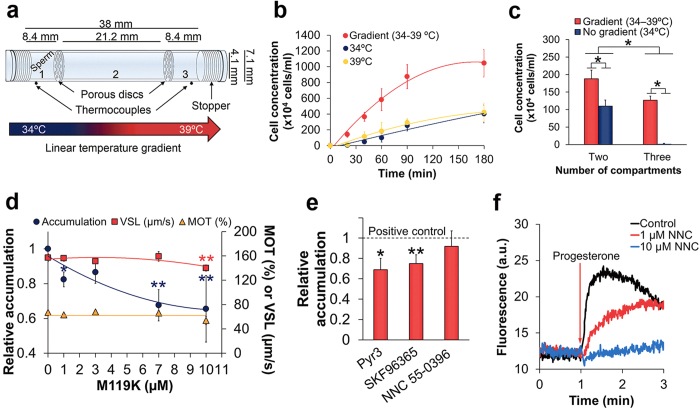
Involvement of G-protein βγ and TRPCs in human sperm thermotaxis. (**a**) Illustration of the thermoseparation tube. Compartment #1 was filled with spermatozoa in capacitating medium (70 × 10^6^ cells/ml), and the other two—with the medium only. (**b**) Time-dependent sperm accumulation in compartment #3 when the tube is in a linear temperature gradient (34–39 °C) or in a constant, uniform temperature (no-gradient control at 34 °C or 39 °C). Each result is the mean ± SEM of 3 determinations. (**c**) Comparison between sperm accumulation (mean ± SEM; n = 12) in the warmest compartments of two- and three-compartment tubes following the separation period (15 and 20 min, respectively). The accumulations comprised 7.5 ± 1.0% and 4.4 ± 0.7% of the total number of spermatozoa with and without the gradient in the two-compartment tube, respectively, and 1.8 ± 0.2% and 0.013 ± 0.001% in the three-compartment tube. *P < 0.01 according to paired two-tailed Student’s *t*-test (for the difference between the gradient and no-gradient control in the two-compartment tube), Wicoxon Signed Ranks test (for this difference in the three-compartment tube), and unpaired two-tailed Student’s *t*-test (for the difference between these two differences). (**d**) Effects of M119K on the relative sperm accumulation, VSL, and percentage of motile cells (MOT). The absolute value of sperm accumulation in the warmer compartment (#3) in the absence of M119K (defined as 1) was (122 ± 11)×10^4^ cells/ml (mean ± SEM of 9–18 determinations). *P < 0.02, **P ≤ 0.002 according to two-way ANOVA. The slope of the relative-accumulation curve was significantly different from the other two slopes (*P* < 0.001 according to three-way ANOVA). The absolute value of the no-gradient accumulation in compartment #3 was (2.0 ± 0.3)×10^4^ cells/ml independently of whether M119K was present or not. (**e**) Effects of Pyr3 (1 μM), SKF96365 (1 μM) and NNC (10 μM) on sperm thermotaxis, as reflected in the relative accumulation in the warmer compartment. The absolute values of sperm accumulation in the absence of the inhibitors were (144 ± 27)×10^4^, (146 ± 11)×10^4^ and (149 ± 26)×10^4^ cells/ml for Pyr3, SKF96365 and NNC (mean ± SEM of 9, 13 and 18 determinations, respectively). *P = 0.02, **P = 0.001 according to two-way ANOVA. The no-gradient accumulation was (1–4)×10^4^ cells/ml independently of whether the inhibitors were present or not. (**f**) Inhibition of CatSper by NNC. A representative plot of a population assessment showing Ca^2 + ^intracellular levels in human spermatozoa as fluorescence of the Ca^2 + ^-sensitive dye Fluo-3. Prior to the measurements the relevant samples were incubated for 5 min with NNC at the indicated concentrations. The progesterone concentration was 1 μM.

Our earlier studies demonstrated that human sperm thermotaxis is mediated by phospholipase C (PLC)^8^. Since PLC is a key enzyme in one of the signalling pathways of G-protein-coupled receptors (GPCRs)^9^, this pointed to the possible involvement of GPCRs in thermosensing for thermotaxis. GPCRs are known to signal via the α subunit and βγ complexes of G-proteins^9^,^10^. PLC is typically activated by the G_αq_ subunit^9^ but it can also be activated by G_βγ_^10^. Since there is no commercially available inhibitor of G_αq_, we examined the possibility of GPCR involvement in thermotaxis by studying the effect of M119K, an inhibitor of G_βγ_, on human sperm thermotaxis. To this end we first established a sensitive bioassay for thermotaxis by placing spermatozoa in a three-compartment separation tube (Fig. 1a) within a thermoseparation device that maintains a linear temperature gradient^7^,^8^. We followed the time course of sperm accumulation in the warmer compartment (compartment #3 in Fig. 1a) for up to 3h. Throughout this time the difference between the gradient and no-gradient control was maintained (Fig. 1b). Consistent with Bahat *et al.*^7^, the absolute temperature had very little effect, if any, on the no-gradient accumulation (Fig. 1b). We chose a separation time of 20min, at which the accumulation was sufficiently high in the presence of the gradient and negligible in its absence (Fig. 1b and right columns in Fig. 1c). The assay thus provided a relatively large difference between the experiment and control. This large difference is unprecedented in accumulation assays with mammalian spermatozoa because in earlier accumulation assays, which employed two-compartment separation tubes^7^,^8^, the no-gradient control was substantially higher than in the three-compartment tubes, resulting in much smaller relative difference (Fig. 1c, left columns). Hereafter we defined the ratio between the gradient-dependent accumulations in the warmer compartment in the presence and absence of M119K (or any other studied effector) as ‘relative accumulation’. Because this definition assumes that the effector does not affect the no-gradient accumulation, we experimentally verified this assumption for each tested effector. Treating human spermatozoa with increasing concentrations of M119K reduced the relative accumulation in a dose-dependent manner without affecting the no-gradient accumulation, the linear swimming speed (termed straight-line velocity, VSL) and, excluding at the highest tested concentration, the fraction of motile cells (Fig. 1d). The observed inhibition of thermotaxis was partial probably because, as mentioned above, the G_βγ_ pathway is not the only one involved in PLC signalling^9^. We verified that M119K indeed did not affect the no-gradient sperm accumulation by repeating the experiments in the two-compartment separation tube, where the no-gradient accumulation is higher and, therefore, negative effects of the drug, if exist, are more easily measureable. Testing the whole range of M119K shown in Fig. 1d, there was no difference in the no-gradient accumulation between the presence and absence of the drug (blue columns in Supplementary Fig. S1 online), similarly to the findings made with the three-compartment tube. The inhibition of the gradient-dependent accumulation but not of the no-gradient accumulation by M119K suggested the involvement of GPCRs in thermotaxis.

Since GPCRs that signal through the activation of PLC are known to lead to the opening of TRPC channels^11^, we examined whether these channels, found to exist in human spermatozoa^12^, are involved in sperm thermotaxis. Specifically, we examined the effects of the TRPC inhibitor SKF96365^12^ and the TRPC3 inhibitor Pyr3^13^ on sperm thermotaxis, each used at a concentration comparable or lower than those in the literature^12^,^13^. Both inhibitors significantly reduced the number of cells accumulating in the warmer compartment of the three-compartment thermoseparation tube (Fig. 1e) without affecting the motility (Table 1) or the no-gradient accumulation. This suggested the involvement of TRPC3, and perhaps additional TRPC channels, in sperm thermotaxis.

Thermotaxis can be mediated by chemotaxis receptors in some systems, e.g., in *Escherichia coli*^1^,^14^. This does not seem to be the case in human sperm thermotaxis because saturating concentrations of chemoattractants, expected to render the receptors insensitive to further stimulation, do not affect the thermotactic response^8^. To verify that chemotaxis receptors are not involved in sperm thermotaxis, we inhibited CatSper—a sperm-specific Ca^2+^ channel known to act, perhaps in association with another protein, as a receptor for the chemoattractant progesterone^15^,^16^. We employed NNC 55–0396 (abbreviated hereafter NNC), a T-type channel blocker shown to be a CatSper blocker in human spermatozoa^15^,^16^. An NNC concentration (10 μM), which fully inhibited progesterone-stimulated Ca^2+^ influx (Fig. 1f), neither affected sperm accumulation in the warmer compartment (Fig. 1e) nor the two motility parameters on which the sperm accumulation in the warmer compartment is expected to be linearly dependent—the linear speed and percentage of motile cells (bold columns in Table 1). The inhibition of CatSper by NNC and the lack of effect of this inhibition on thermotaxis confirmed that CatSper is not involved in sperm thermotaxis. Taken together, the above results suggest the involvement of GPCRs in thermotaxis. A GPCR shown to have thermosensing capability is rhodopsin, demonstrated in thermotaxis of *Drosophila* larvae^17^. Therefore, we examined whether this GPCR and other opsins are present in human spermatozoa.

### Opsin mRNAs are present in sperm

**Figure 2:**
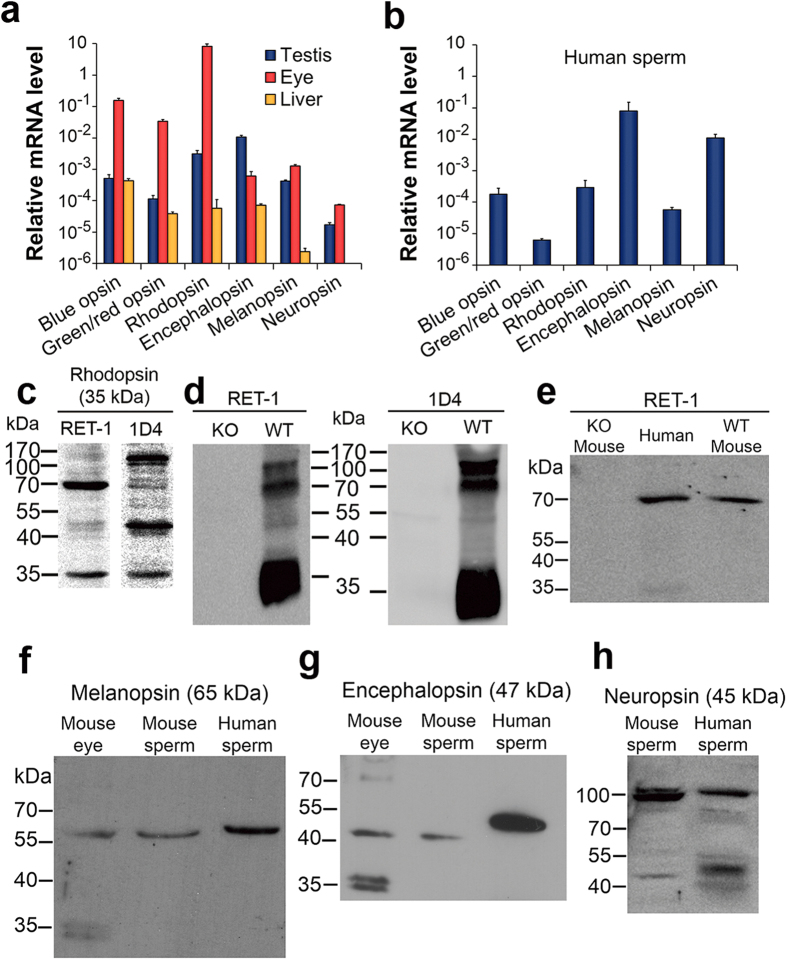
Presence of opsins in mammalian spermatozoa. (**a**) Quantitative real-time PCR analysis of opsin mRNAs in mouse testes and liver. Mouse eyes were used as a positive control. (**b**) Quantitative real–time PCR analysis of opsin mRNAs in mature human spermatozoa. (**c**) A representative Western-blot analysis of rhodopsin in human spermatozoa using the monoclonal antibodies RET-1 and 1D4. Each lane contained 15 μg protein in total. (**d**) A representative Western-blot analysis of rhodopsin in eyes from rhodopsin-knockout (KO) and wild-type (WT) mice. Rhodopsin was extracted with the same lysis buffer used for human spermatozoa (see *Methods*). Each lane contained 15 μg protein in total. (**e**) A representative Western-blot analysis of rhodopsin in human and mouse spermatozoa using the monoclonal antibody RET-1. The lanes of the wild-type (WT) and rhodopsin-knockout (KO) mouse spermatozoa contained 30 μg protein in total, and the lane of human spermatozoa contained 15 μg protein in total. (**f**) A representative Western-blot analysis of melanopsin in mouse eye and spermatozoa and in human spermatozoa. The lane of the eye contained 5 μg protein in total and the lanes of mouse and human spermatozoa contained 15 μg protein each. (**g**) A representative Western-blot analysis of encephalopsin in mouse eye and spermatozoa and in human spermatozoa. The lane of the eye contained 5 μg protein in total and the lanes of mouse and human spermatozoa contained 15 μg protein each. (**h**) A representative Western-blot analysis of neuropsin in human and mouse spermatozoa. Each lane contained 15 μg protein in total.

Although earlier studies detected mRNAs encoding encephalopsin (Opsin-3)^18^ and neuropsin (Opsin-5)^19^ in mouse testes, no reports on the detection of mRNAs of rhodopsin and other opsins in the testis, or on the expression of any opsin proteins in the testis, were available. To determine whether opsins are expressed in mammalian spermatozoa, we first looked for the presence of opsin-encoding mRNAs in mouse testes by quantitative real-time PCR (Fig. 2a). The mRNAs of rhodopsin and encephalopsin were the most abundant, followed by melanopsin (Opsin-4) and blue opsin (Short-wave-sensitive opsin 1). As a positive control, we compared these levels to those in the mouse eye. As might be expected, the mRNA levels of all visual opsins [rhodopsin, blue opsin, green opsin (Medium-wave-sensitive opsin 1), and red opsin (Long-wave-sensitive opsin 1)] in the eye were orders of magnitude higher than those in the testis (note the logarithmic scale), but the levels of the other opsins were comparable in both tissues. Encephalopsin was even an order of magnitude higher in the testis than in the eye (Fig. 2a). The mouse liver, known to contain encephalopsin but not neuropsin^20^,^21^ and tested as another control, indeed contained mRNA of encephalopsin and not of neuropsin. Yet, it also contained mRNAs of the visual opsins and, to a much lesser extent, melanopsin (Fig. 2a). Analysis of human spermatozoa revealed mRNAs of all the seven studied opsins, with encephalopsin and neuropsin being the most abundant (Fig. 2b).

### Opsin proteins are present in human sperm

To establish the presence of opsin proteins in spermatozoa, we first employed Western blots, using two different anti-rhodopsin antibodies. The two monoclonal antibodies, RET-1 against the *N*-terminus of rhodopsin^22^ and 1D4 against the *C*-terminus^23^, identified the same bands at the expected molecular sizes of monomeric and dimeric rhodopsin (35 and 70 kDa, though with different relative intensities; Fig. 2c), indicating that rhodopsin is indeed present in human spermatozoa. The additional bands at 40 and 48 kDa are probably the outcome of different levels of glycosylation^24^. The high molecular-mass band likely reflects rhodopsin tetramer^25^. To confirm the specificity of both antibodies, we used them in Western-blot analysis of protein extracts of eyes obtained from wild-type and rhodopsin-knockout mice. Both antibodies detected bands at the sizes of monomeric and dimeric rhodopsin as well as presumably higher molecular weight oligomers in the wild-type extract but not in the knockout eye extract (Fig. 2d). We also detected a band of dimeric rhodopsin in mouse spermatozoa; this band was absent in spermatozoa of rhodopsin-knockout mice (Fig. 2e). The absence of the bands in eyes and spermatozoa of rhodopsin-knockout mice confirm the specificity of the antibodies and the presence of rhodopsin in mature human and mouse spermatozoa.

To determine whether additional opsins are present in human and mouse spermatozoa, we employed specific antibodies against three other opsins. Antibodies against melanopsin (Fig. 2f) and encephalopsin (Fig. 2g) detected bands that correspond to the monomeric forms of these opsins both in human and mouse spermatozoa as well as in mouse eyes, used as a positive control. The anti-neuropsin antibody detected a band at the expected molecular size of the monomeric and dimeric forms of this opsin both in mouse and human spermatozoa (Fig. 2h). These bands were not detected when the antibody was used in the presence of its specific blocking peptide, confirming the specificity of the antibody. Blocking peptides for the anti-melanopsin and anti-encephalopsin antibodies used herein are commercially unavailable. These results suggest that other opsins, additional to rhodopsin, are present in human and mouse spermatozoa.

### Opsin proteins are present at specific locations in spermatozoa

**Figure 3:**
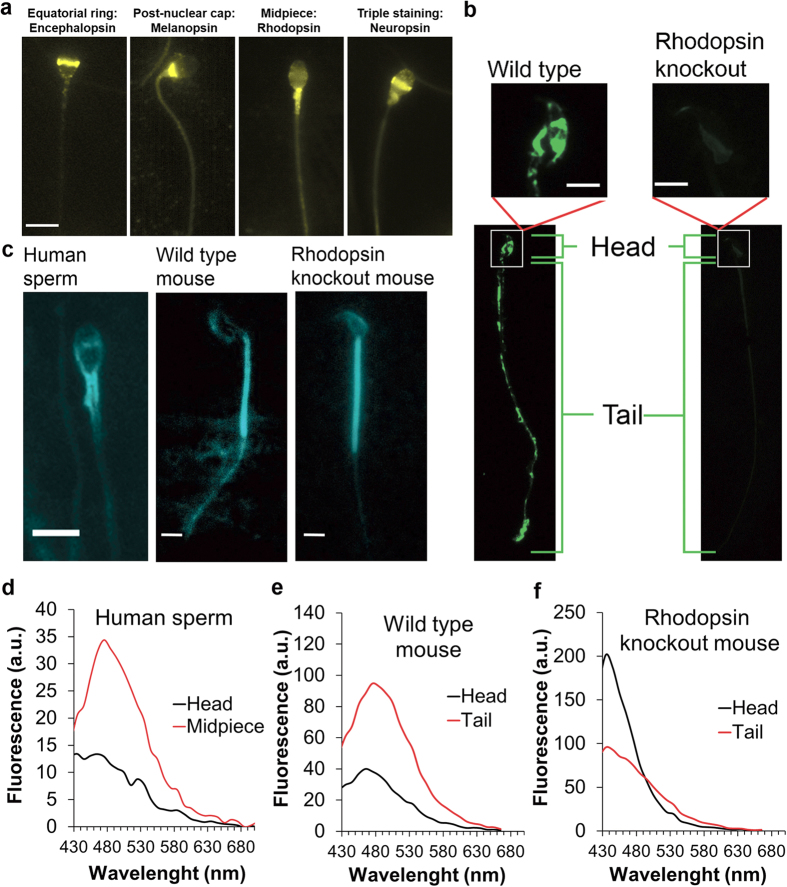
Localization of rhodopsin and retinal in mammalian spermatozoa. (**a**) Representative images showing the different locations of opsins in human spermatozoa, revealed by immunocytochemical analysis. The anti-rhodopsin antibody was H-300. Bar = 5 μm. (**b**) Representative immunocytochemical staining of rhodopsin in mouse spermatozoa retrieved from wild-type and rhodopsin-knockout mice. Bar = 5 μm. **(c)** Representative TIRF images of human and mouse spermatozoa. Excitation was at 473 nm and emission at 535 ± 15 nm. Bar = 5 μm. (**d**) Emission spectra of the head and midpiece of human spermatozoa obtained by confocal microscopy (Ex = 406 nm). The spectra are the averages of 30 heads and 25 midpieces. **(e)** Emission spectra of the head and tail of wild-type mouse spermatozoa obtained by confocal microscopy (Ex = 406 nm). The spectra are the averages of 14 heads and 20 tails. The tails included the midpiece. (**f**) Emission spectra as in panel e but from rhodopsin-knockout mice. The spectra are the averages of 15 heads and 22 tails.

To further test this possibility we employed immunocytochemical analysis of human spermatozoa. All the opsins examined seemed to be at one or more of the following locations: the equatorial ring in the sperm head, the postnuclear cap, and the midpiece (Fig. 3a for representative images; Supplementary Table S1 online for location distributions). Notably, PLC signalling components (the G-protein α_q/11_ subunit^26^, PLC^27^, the inositol 1,4,5-trisphosphate receptor^28^, the Ca^2+^ store^29^, and some TRPCs, including TRPC3^12^) also reside in one or more of these specific locations. Immunocytochemical analysis of wild-type and rhodopsin-knockout mouse spermatozoa, carried out as a control with the anti-rhodopsin monoclonal antibody RET-1, revealed staining in the wild-type cells only (Fig. 3b). Unlike in human spermatozoa (Fig. 3a), the staining in wild-type mouse spermatozoa was mainly in patches all along the tail (Fig. 3b), suggesting different distributions of opsins in human and mouse spermatozoa. This is not surprising in view of their so different morphologies.

**Figure 4:**
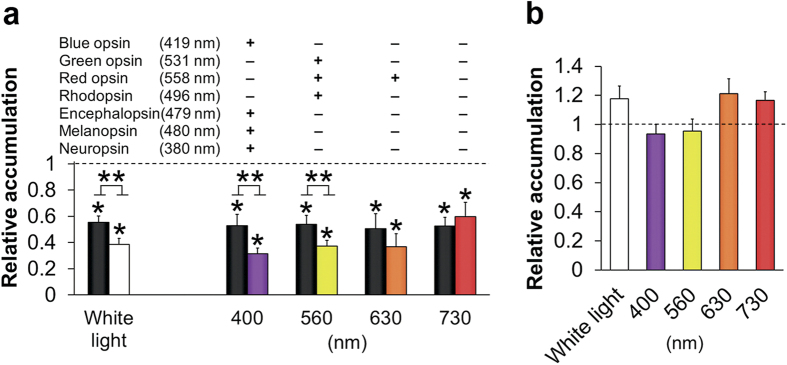
Effects of hydroxylamine and light on human sperm thermotaxis. (**a**) Effect of hydroxylamine on sperm thermotaxis, as reflected in the relative accumulation in the warmer compartment. Prior to thermoseparation, the spermatozoa were incubated with hydroxylamine (5 mM) for 30 min at room temperature in the dark (black column) or under illumination (white/color columns). The plus or minus signs above the columns stand for whether or not the indicated opsin absorbs at the wavelength of the illumination ( ≥ 25% of the absorbance at its peak). Each experimental result is the mean ± SEM of 5–9 determinations. The positive control, defined as 1, is the sperm accumulation in the absence of hydroxylamine in the dark [being almost the same in each set of experiments, (140 ± 8)×10^4^cells/ml (mean ± SEM of 5–12 determinations)]. *P (relative to the positive control in the dark) and **P (light *versus* dark in each wavelength in the presence of hydroxylamine) < 0.03 according to two-way ANOVA. The no-gradient accumulation was (1.6 ± 0.2)×10^4^cells/ml independently of whether hydroxylamine was present or not. (**b**) Effect of illumination on thermotaxis in the absence of hydroxylamine. Spermatozoa were incubated for 30 min at room temperature in the dark or under illumination at the indicated wavelength prior to thermoseparation. Each experimental result is the mean ± SEM of 7–10 determinations. The dark control was defined as 1 [(141 ± 8)×10^4^ cells/ml (5–13 determinations)]. The results were not significantly different from the control (two-way ANOVA).

All opsins are known to be bound via a Schiff base at a conserved lysine residue (Lys–296 in rhodopsin) to the aldehyde form of vitamin A, retinal^30^. This chromophore has an essential role in the photoresponse of opsins. In the case of human opsins, it undergoes light-stimulated isomerization from 11-*cis*-retinal to all *trans*-retinal, and the protein undergoes a conformational change and interacts with the G-protein transducin^30^. Although rhodopsin itself, and presumably other opsins as well, are not detectably fluorescent, fluorescence emission from some of the intermediates of their photocycle (e.g., metarhodopsin) is detectable^31^. If opsins in sperm are activated by temperature, it is reasonable to assume that fluorescent intermediates might be detected even in the dark. We, therefore, examined whether human and mouse spermatozoa are fluorescent and, if so, whether this fluorescence is related to opsins, at least partially. To restrict the measured fluorescence to the cell membrane, we employed total internal reflection fluorescence (TIRF) microscopy, only detecting the fluorescence emitted from the cell surface touching the microscope slide. We detected fluorescence at the locations identified earlier by immunocytochemistry to contain opsins (Fig. 3c; cf. Fig. 3a,b). If this fluorescence is linked, at least in part, to opsins, we expected it to be affected by the absence of rhodopsin in knockout mice. We detected no significant difference between the internal fluorescence intensities of the wild-type and rhodopsin-knockout spermatozoa probably because of the presence of opsins other than rhodopsin and, perhaps, additional fluorophores. However, the emission spectra in both the sperm head and tail, measured by confocal microscopy were blue-shifted (Fig. 3d,e *versus* Fig. 3f), suggesting that retinal-opsins contributed to the measured fluorescence. Nevertheless, using all *trans*-retinal and 11-*cis* retinal as standards, we did not detect these two compounds in human spermatozoa by liquid chromatography with tandem mass spectrometry (LC-MS/MS with a detection limit of 10^−15^ moles). This fact taken with the presence of retinal precursors in spermatozoa^32^ and with the results, shown below (Fig. 4), indicating the functional presence of retinal in sperm, suggests that retinoids different from all *trans* and 11-*cis* retinal, or a different fluorophore altogether, are involved in thermosensing. Consistently, when we measured the effect of the intensity and wavelength of illumination on the motility, we found that all the studied parameters were unaffected, excluding a minor change in VCL and, consequently, in LIN (Table S2). This minor change could well be the outcome of the light-dependent changes in the contrast of the images and their consequent effect on sperm tracking. The cells also did not exhibit phototaxis to, or away from, blue light (480 nm wide-band interference filter), to which most of the opsins are sensitive^21^. All these results suggest that the retinoid associated with sperm opsins is involved in temperature sensing rather than photosensing.

### Opsins are involved in human sperm thermotaxis

The nucleophile hydroxylamine is known to attack the Schiff base between opsin and retinal and inhibit the opsin function, but the sensitivities of the various opsins to this drug are different. Thus, rhodopsin is inhibited by hydroxylamine in the light only^33^, whereas blue, green, and red opsins are inhibited in both light and dark^30^. In contrast, melanopsin is resistant to hydroxylamine^34^. To test for the functional involvement of opsins in thermotaxis, we took advantage of these facts. We studied the dose-dependence of hydroxylamine on thermotaxis and chose to work with 5 mM—the lowest concentration that, while having a maximal effect on thermotaxis, had a minimal effect on motility. This concentration is orders of magnitude lower than in many published studies, including studies with systems of highly accessible rhodopsin^33^,^34^,^35^,^36^. We incubated human spermatozoa with hydroxylamine prior to, and during the assay, and examined its effect on the thermotactic response. Incubation with hydroxylamine in the dark significantly reduced sperm accumulation in the warmer compartment of the separation tube without affecting the no-gradient accumulation (Fig. 4a, black columns). This reduction could be the outcome of speed decrease (Table 1). However, the speed reduction as reflected in VSL, on which the sperm accumulation in the warmer compartment is expected to be linearly dependent, was 16% only, whereas sperm accumulation in the dark was 46% inhibited, suggesting that some of the reduced accumulation was the outcome of thermotaxis inhibition. When the incubation with the drug prior to the assay was performed under illumination (the assay itself was carried out in the dark because of being within the metallic thermoseparation device), additional inhibition of thermotaxis was observed (Fig. 4a, White light). This additional light-dependent inhibition by hydroxylamine was not accompanied by speed reduction (Table 1). The same illumination prior to the assay but in the absence of hydroxylamine had no significant effect on either sperm accumulation (Fig. 4b, White light) or sperm motility (Table 1). The inhibition of thermotaxis by hydroxylamine was, as expected, partial due, at least in part, to the fact that not all opsins are sensitive to this drug.

Since thermotaxis is carried out by modulating the frequency of hyperactivation events^5^, we expected that if, indeed, opsins act as temperature sensors in thermotaxis, their inhibition by hydroxylamine may inhibit their ability to signal to the flagellum and, consequently, inhibit the resulting changes in hyperactivation. We, therefore, examined the effect of hydroxylamine in the light on temperature-stimulated changes in hyperactivation. Hydroxylamine had two effects. It significantly reduced the level of hyperactivation, reflected in the percentage of hyperactivated spermatozoa and in the fractal dimension (FD, which is a quantitative measure of the intensity of hyperactivation^37^), and it prevented the temperature-stimulated changes in hyperactivation (Table 2). These results endorse the involvement of opsins in thermotaxis.

Each opsin is sensitive to a different wavelength range^21^. To obtain an indication as to whether different opsins are involved in thermotaxis, we carried out thermotaxis assays following incubation with hydroxylamine under illumination at specific wavelengths. Hydroxylamine added with 400 nm laser illumination (which excites blue opsin, encephalopsin, melanopsin and neuropsin) or with 560 nm laser illumination (which excites rhodopsin as well as green and red opsin) significantly reduced sperm accumulation (Fig. 4a, right columns) without affecting the motility relatively to hydroxylamine added in the dark (Table 1). Apparently, illumination made the Schiff base more accessible to hydroxylamine^34^. Hydroxylamine added with 630 nm illumination (which excites red opsin) appeared to have a similar inhibitory effect, but this effect was statistically insignificant. The observation that two different groups of opsins responded to wavelengths that excite different opsins suggests that more than one opsin type is involved in sperm thermotaxis. Illumination at 730 nm (which excites none of the opsins) had no significant effect on either sperm accumulation (Fig. 4a) or sperm motility (Table 1). None of the laser illuminations *per se* affected the sperm accumulation (Fig. 4b) or motility (Table 1). Consistently with the quantitative real-time PCR, Western blot and immunocytochemistry, these results suggest that several different opsins are involved in thermotaxis. The wavelength-dependent inhibition of thermotaxis by hydroxylamine further suggests that retinal, in a form yet unidentified, may be required for thermosensing in sperm thermotaxis.

### Thermotaxis is defective in rhodopsin-knockout mice

**Figure 5:**
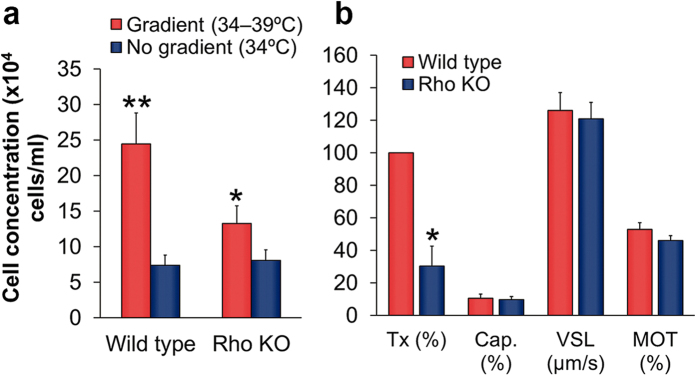
Sperm thermotaxis, capacitation and motility of rhodopsin-knockout mice. (**a**) Gradient-dependent sperm accumulation of wild-type and knockout mice. The columns stand for the accumulation of spermatozoa in the warmer compartment, using the two-compartment separation tube (mean ± SEM of 9 wild-type and 11 rhodopsin-knockout mice). *P = 0.03 and **P = 0.009 according to repeated measures ANOVA. (**b**) Comparison between wild-type and knockout mice with respect to sperm thermotaxis, capacitation and motility. Thermotaxis was defined as the difference between the gradient and no-gradient columns in panel A. This difference for the wild-type spermatozoa was defined as 100%, and the difference found with the spermatozoa of the knockout mice was calculated relatively to this value [Tx (%)]. *P < 0.001 according to repeated measures ANOVA. The values shown for capacitation (Cap.) are the mean ± SEM of 5 determinations for each strain (500–800 cells in each determination). The values shown for VSL and for the percentage of motile cells [MOT (%)] are the mean ± SEM of 25 and 28 determinations for wild-type and knockout mice, respectively (300–1800 cells per determination). These parameters in the spermatozoa from the knockout mice were not significantly different from those from the wild-type mice (unpaired two-tailed Student’s t test).

To further examine the involvement of opsins in sperm thermotaxis we used rhodopsin-knockout mice and compared their sperm thermotaxis with that of wild-type mice. Since the existence of sperm thermotaxis in mice had not been studied, we first established its occurrence and a protocol to measure it. With the limited number of spermatozoa that can be isolated from the mouse epididymis, we employed two-compartment separation tubes, described earlier^7^,^8^, which only require 5 × 10^6^ cells/ml (*versus* 70 × 10^6^ cells/ml in the three-compartment tube described above—Fig. 1a). The accumulation of mouse spermatozoa in the warmer side of the separation tube was significantly higher in the presence of a temperature gradient than in its absence (Fig. 5a, left columns), confirming the occurrence of mouse sperm thermotaxis. As in human and rabbit spermatozoa^3^, the fraction of responsive cells was small, 1–8%, probably reflecting the relatively low fraction of capacitated cells—the only ones that have the capacity to respond thermotactically in a spatial temperature gradient^3^ and to fertilize an egg^38^. We then compared between wild-type and knockout spermatozoa with respect to thermotaxis and parameters that can affect sperm accumulation in the warmer compartment: the fraction of motile cells, their linear velocity, and the level of capacitated cells, defined herein as the level of A23187-induced acrosome-reacted cells. Since the level of capacitation is a reflection of the sperm ability to fertilize an oocyte^38^ and since capacitation involves many biochemical and signalling processes^39^, the capacitation level is an excellent control for the overall signalling and physiological processes in the spermatozoa. The level of capacitated cells in the knockout spermatozoa was as in the wild-type cells, and so were the fraction of motile cells and their linear velocity, indicating that the knockout spermatozoa are as wild-type spermatozoa with respect to physiology and signalling (Fig. 5b). Nevertheless, thermotaxis, reflected in sperm accumulation in the warmer compartment, was 70% reduced in the knockout cells when measured in a temperature gradient (Fig. 5). In contrast, the no-gradient accumulation of knockout spermatozoa was similar to that of wild-type cells (Fig. 5a). The residual thermotactic activity in the knockout cells was expected because opsins other than rhodopsin were still present in these cells. These observations substantiate the involvement of rhodopsin in sperm thermotaxis.

We are aware of the findings that the response of human and mouse spermatozoa to the chemoattractant progesterone is different, suggesting differences between these species^16^. Yet, the similarity between mouse and human spermatozoa with respect to the results and conclusions of this study suggests that, as far as thermotaxis is concerned, mouse and human spermatozoa are similar.

### At least two signalling pathways are involved in sperm thermotaxis

**Figure 6:**
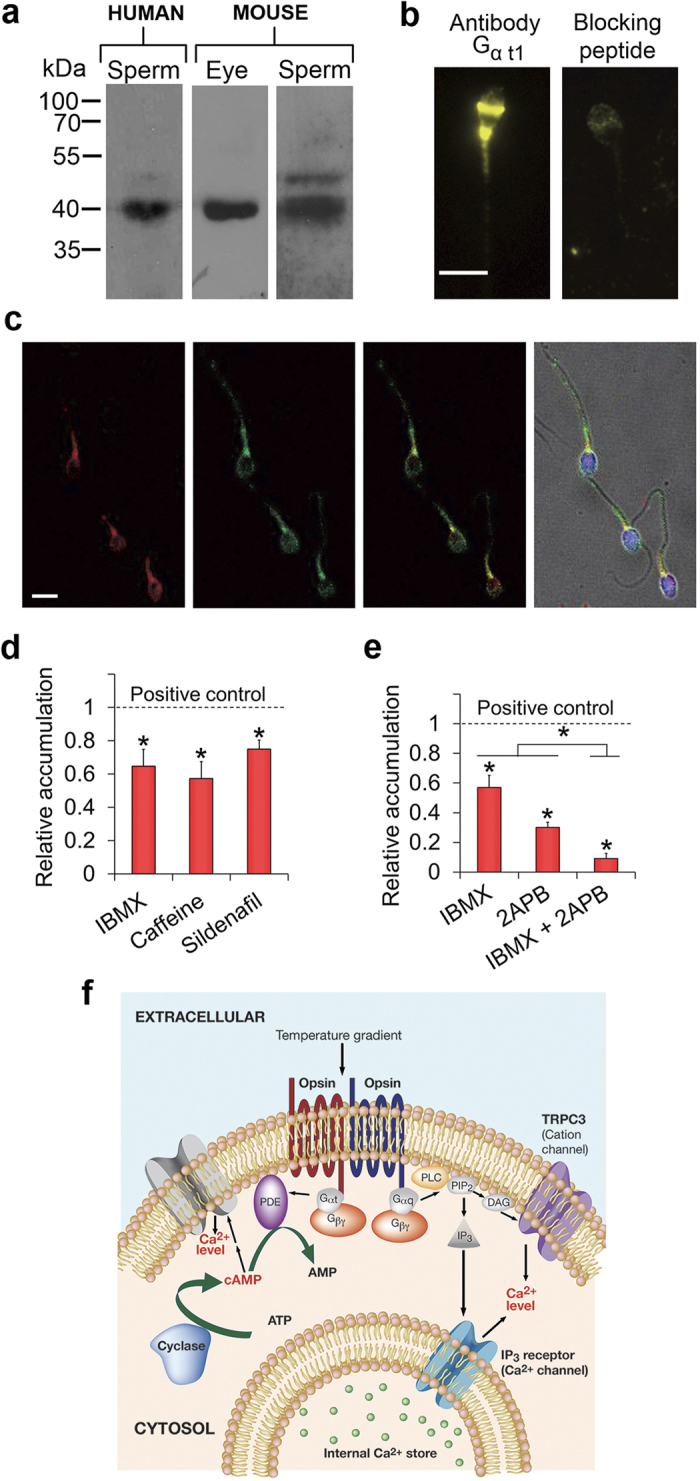
Involvement of cyclic-nucleotide signalling in sperm thermotaxis. (**a**) A representative Western-blot analysis of G_α t1_ in human and mouse spermatozoa. Mouse eyes were used as a positive control. (**b**) Localization of G_α t1_ in human spermatozoa by immunocytochemical analysis. The right panel is a negative control in which the cells were incubated with both the primary antibody and a specific blocking peptide. Bar = 5 μm. (**c**) Representative images showing co-localization of G_α t1_ and rhodopsin in human spermatozoa, revealed by immunocytochemical analysis. From left to right: G_α t1_ (red), rhodopsin (green), G _α t1_ and rhodopsin together (yellow), nuclei stained with TO-PRO^®^–3 (blue) overlaid with the double-staining on the bright-field image. No staining was observed in a negative control in which the cells were incubated with the primary antibodies and their specific blocking peptides together. Bar = 5 μm. (**d**) Effects of PDE inhibitors on sperm thermotaxis, as reflected in the relative accumulation in the warmer compartment. The inhibitors were IBMX (1 mM), caffeine (2 mM), and sildenafil (100 nM). The absolute values of sperm accumulation in the absence of the inhibitors were (165 ± 12)×10^4^, (174 ± 19)×10^4^ and (222 ± 17)×10^4^cells/ml for IBMX, caffeine and sildenafil (mean ± SEM of 13, 16 and 18 determinations, respectively). *P ≤ 0.02 according to two-way ANOVA. The absolute value of the no-gradient accumulation was independent of the presence of inhibitors and ranged between (1–4)×10^4^ cells/ml. (**e**) Effects of PDE and PLC inhibitors on sperm thermotaxis, as reflected in the relative accumulation in the warmer compartment. The inhibitors were IBMX (1 mM) and 2APB (0.1 mM). The absolute values of sperm accumulation in the absence of the inhibitors was (136 ± 10)×10^4^ (mean ± SEM of 10 determinations). *P ≤ 0.05 according to three-way ANOVA. The absolute value of the no-gradient accumulation was independent of the presence of inhibitors and ranged between (10–17)×10^4^ cells/ml. Since this value was not negligible, we subtracted it from the gradient accumulation values prior to calculating the relative accumulation. (**f**) A model for two signalling pathways in sperm thermotaxis. The PLC pathway is shown on the right, and the cyclic-nucleotide pathway is shown on the left. See Supplementary Discussion for details. Coloured and grey objects stand for signalling components for which direct evidence exists or is not yet available, respectively.

Of the visual opsins, only melanopsin is known to signal via PLC^30^. Blue, green and red opsins as well as rhodopsin signal via transducin and cyclic nucleotides^40^. This also seems to be the case for encephalopsin^41^ and neuropsin^21^. To substantiate our conclusion that several different opsins are involved in sperm thermotaxis, we examined whether, as in vision, the cyclic-nucleotide pathway, additionally to the PLC pathway^8^, is involved in thermotaxis. If it is involved, one expects to find that transducin is present in sperm, that phosphodiesterase (PDE), known to regulate together with adenylyl or guanylyl cyclase the intracellular level of cyclic nucleotides^42^, is involved in thermotaxis, and that a temperature shift stimulates changes in the intracellular level of a cyclic nucleotide. We restricted ourselves to these parameters, as revealing the molecular mechanism underlying thermotaxis is a topic of a study of its own. We first examined by Western blots whether human spermatozoa at all contain the G_i_ protein transducin-1, known to be activated by rhodopsin in vision^40^. A monoclonal antibody against the alpha subunit of transducin-1 (G_α t1_) revealed a band at the expected molecular size (40 kDa) in human and mouse spermatozoa and, as a positive control, in mouse eyes (Fig. 6a). Immunocytochemical analysis with a polyclonal anti-G_α t1_ (K-20) antibody indicated that the location of G_α t1_ is in the equatorial ring, the postnuclear cap and the mid-piece (Fig. 6b and Supplementary Table S1 online)—the same locations where opsins were found to reside. The antibody did not stain any of these regions in the presence of its blocking peptide, demonstrating its specificity. To verify that, indeed, transducin-1 and rhodopsin co-localise in sperm, we employed double staining with antibodies against these two proteins. The immunocytochemical double-staining clearly showed co-localisation of both proteins (Fig. 6c). Transducin-2, the G-protein that is activated in vision by blue, green and red opsins, was reported to exist in human spermatozoa as well^43^.

To determine whether PDE is involved in thermotaxis, we studied the effects of the general PDE inhibitors caffeine and 3-isobutyl-1-methylxanthine (IBMX) and the specific PDE5/6 inhibitor sildenafil^44^ on human sperm thermotaxis, expecting to see partial inhibition if the cyclic-nucleotide pathway is involved in parallel to the PLC pathway. Each of these three inhibitors partially inhibited thermotaxis (Fig. 6c); they neither inhibited the no-gradient sperm accumulation nor the swimming speed, and they did not reduce the fraction of motile cells (Table 1), supporting the involvement of a cyclic-nucleotide signalling pathway in thermotaxis.

Finally, we looked for changes in the intracellular concentrations of cAMP and cGMP in response to a fast temperature shift. We divided each sperm sample to two portions, one incubated for 15 min at 34 °C and one at 39 °C. Subsequently we quickly shifted each of them to the other temperature and measured the levels of their cyclic nucleotides as a function of time. While the level of cGMP remained essentially unchanged within our time resolution, the level of cAMP transiently increased and decreased when the temperature was shifted up and down, respectively (Table 3). It should be pointed out that 1 s was our first measureable time point after the zero point. It is not impossible that, as in sperm chemotaxis^45^, changes in the level of cGMP, too fast to be detected by the method employed in this study, occur as well.

The presence of transducin-1 and -2 in sperm and the colocalization of the former with opsins, the inhibition of thermotaxis by PDE inhibitors, and the detection of temperature-stimulated changes in the intracellular level of cAMP, taken together, suggest the involvement of the cyclic nucleotide-pathway in thermotaxis, additional to the PLC pathway identified earlier^8^. To substantiate the conclusion that both pathways are involved in thermotaxis, we examined the effect of simultaneous inhibition of these pathways on human sperm thermotaxis. Specifically, we examined the effect of the PDE inhibitor IBMX together with the PLC-pathway inhibitor 2-aminoethoxydiphenyl borate (2APB). The latter blocks both SOCs and IP_3_R^46^, but SOCs are not involved in sperm thermotaxis, and 2APB partially inhibits thermotaxis by blocking the IP_3_R Ca^2+^ channel^8^. Both inhibitors together essentially abolished thermotaxis, whereas each of them alone caused partial inhibition only (Fig. 6e). The inhibitors (combined and separately) had no effect on the no-gradient accumulation. The linear speed, VSL, was indeed reduced by 2APB and by both inhibitors together due to increased hyperactivation (FD in Table 1), but the inhibition of thermotaxis was much larger (Fig. 6e). It should be noted that hyperactivation is a major component of the thermotactic response^5^. Therefore, strong inhibitors of thermotaxis are expected to affect the level of hyperactivation and, consequently, the related motility parameters—cf. Table 2. This is also the case in bacterial chemotaxis, where major interferences with the signalling pathway almost always result in enhanced or decreased frequency of tumbling—the *E. coli*’s equivalence of hyperactivation. The almost complete inhibition of the gradient-dependent accumulation but not the no-gradient accumulation by the inhibitors endorse the conclusion that both the cyclic nucleotide and PLC pathways are involved in sperm thermotaxis (Fig. 6f for a model).

## Discussion

The mammalian opsin family contains at least nine opsins, present in the eye and involved in light sensing^30^. Here we studied several of them and showed that they are also present in mammalian spermatozoa and involved in sperm thermotaxis. Given the fact that they are, by definition, receptors (GPCRs) known to act as seven-transmembrane photosensors^30^, we propose that their function in sperm thermotaxis is temperature sensing. Our findings that transducin −1, i.e., the rhodopsin-cognate G protein, resides in the same locations as opsins, and that the cyclic nucleotide-signalling pathway, which includes transducin, is involved in thermotaxis are consistent with the function of opsins as thermosensors. The results obtained in this study combined with our earlier study^8^ suggest that opsins signal to the flagella via at least two signalling pathways, the PLC pathway and the cyclic-nucleotide pathway (Fig. 6f for a model; see Supplementary Discussion). Consistently, inhibition of both pathways resulted in almost total loss of thermotaxis (Fig. 6e). If there is similarity between the visual and sperm systems with respect to the identity of the signalling pathway associated with each opsin, the opsin involved in the PLC pathway is likely melanopsin^30^,^47^, consistent with its location almost exclusively at the postnuclear cap, which is the location of the non-acrosomal Ca^2+^ store involved in PLC signalling^29^. The other opsins are possibly involved in signalling via transducin and cyclic nucleotides, with rhodopsin likely affecting transducin-1, and blue, green and red opsins affecting transducin-2^21,40,41^. More studies are required for fully revealing these pathways, for resolving how the pathways integrate, and for addressing the question of whether the opsin thermosensors can provide the measured extreme temperature sensitivity of sperm thermotaxis. The different distributions of the various opsins in spermatozoa combined with two specific signalling pathways may be an important factor in this sensitivity.

Sperm thermotaxis is considered to be one of the mechanisms of sperm guidance in mammals. It is thought to be a long-range mechanism^6^,^48^. The very small numbers of capacitated spermatozoa in the Fallopian tube and the large dimensions of the tube relative to the dimensions of the gametes indicated that sperm guidance is absolutely essential for mammalian fertilization^48^. Like most other essential processes in biology^49^, mammalian sperm guidance is expected to involve redundancy. Indeed, sperm rheotaxis—a process of orienting and swimming against a flow of the surrounding fluid— has been recently found in mouse and human spermatozoa and proposed to be another long-range guidance mechanism in mammals^50^. It is, therefore, expected that inhibition of one of the signalling pathways of thermotaxis or even complete inhibition of thermotaxis would have little effect, if any, on fecundity. This well explains our observation that the rhodopsin-knockout mice appeared normally fertile, even though their sperm thermotaxis was 70% reduced (Fig. 5). Redundancy of guidance mechanisms seems to be a general phenomenon. For example, the navigation of migrating birds is unaffected when one of the guidance mechanisms is not functional^51^.

In view of the earlier finding of temperature sensing by rhodopsin in thermotaxis of fly larvae^17^, the current study highlights the evolutionary conservation of opsins in thermotaxis. The findings made in this study may even have broader implications because some opsins were reported to be expressed in other, non-photosensitive organs such as brain^18^,^20^, lung^20^, liver^20^, kidney^20^ and skin^52^. The functions of most of these extra-retina opsins are not known. On the basis of our findings it is reasonable to assume that one of the functions of these opsins in non-photosensitive organs is temperature sensing. According to this notion, opsins form a family of bi-functional molecules that have the potential to sense both temperature and light. As such, they represent a case of conservation of sensors across senses—a conservation that may extend beyond mammals.

## Methods

### Antibodies

The anti-neuropsin antibody (Opsin-5 Polyclonal) and its blocking peptide were from Osenses (Keswick, SA, Australia), anti-melanopsin (Polyclonal Aff–Purified) and anti-encephalopsin (Opsin-3 Polyclonal – Aff–Purified) from Acris Antibodies (San Diego, CA), anti-rhodopsin antibodies rhodopsin (H-300; polyclonal) and 1D4 (monoclonal) from Thermo Fisher Scientific (Waltham, MA), and the monoclonal antibodies “Rhodopsin (Opsin) Ab-1 (Clone RET-P1)” and “G_α t1_ Antibody (3)” as well as the polyclonal “G_α t1_ (K-20)” and “Rhodopsin (I-17)” antibodies and their blocking peptides from Santa Cruz Biotechnology (Santa Cruz, CA).

### Human sperm handling, capacitation and treatments

Studies with human spermatozoa were approved by the Bioethics and Embryonic Stem Cell Research Oversight Committee of the Weizmann Institute of Science. The methods were carried out in accordance with the approved guidelines. Human semen samples were obtained from healthy donors after 3 days of sexual abstinence. Informed consent was obtained from each donor. For the analyses of thermotaxis and motility, semen samples with normal sperm density, motility, and morphology (according to WHO guidelines^53^) were allowed to liquefy for 30–60 min at room temperature. For thermotaxis and motility assays, human spermatozoa were separated from the seminal plasma by centrifugation (240 ×g, 10 min, twice) with capacitating medium (Flushing Medium, which includes human serum albumin, HEPES, bicarbonate, glucose, pyruvate and additional salts; MediCult, Jyllinge, Denmark) supplemented with additional human serum albumin (0.3% w/v final concentration). Subsequently, the sperm concentration was adjusted to 70 × 10^6^ cells/ml in the capacitating medium and incubated for 2 h under an atmosphere of 5% CO_2_ at 37 °C for capacitation^54^. In sperm preparations for protein extraction and immunocytochemistry, the spermatozoa were washed with PBS instead of capacitating medium. To verify that the immunocytochemical analysis faithfully reflects the staining of mature spermatozoa, human spermatozoa were also separated from the seminal plasma by the migration–sedimentation technique^55^ using capacitating medium, thus separating mature swimming cells. Using anti-rhodopsin and anti-encephalopsin antibodies as markers, there was no difference between the two separation methods from the seminal plasma with respect to the distribution of opsins in human spermatozoa.

Incubations with the inhibitors (M119K, SKF96365, Pyr3, caffeine, IBMX, sildenafil, 2APB and hydroxylamine) were performed after capacitation. M119K was a gift from the Developmental Therapeutics Program of the National Cancer Institute, Bethesda, MD. Human sperm samples were incubated with each inhibitor under an atmosphere of 5% CO_2_ at 37 °C for 5 min prior to thermotaxis or motility assays. Stock M119K, Pyr3, IBMX, sildenafil and 2APB were dissolved in DMSO whereas SKF96365 and caffeine were dissolved in H_2_O. In the negative controls, DMSO or H_2_O was added to a final concentration of 1%, like its concentration in the samples treated with the inhibitors. After each treatment the pH was confirmed to be 7.5. Hydroxylamine powder was dissolved directly in capacitating medium and the pH was adjusted to 7.5 prior to its addition to the sample.

In the experiments with hydroxylamine, spermatozoa (500μl at 70 × 10^6^ cells/ml) with or without 5 mM hydroxylamine were placed in 5 ml polystyrene Falcon^TM^ flow-cytometry tubes (BD Bioscience, San Jose, CA) and incubated for 30 min at room temperature in the dark or under illumination, employing a LED flashlight or a laser beam illuminating one side of the tube, and the other side layered with aluminium foil to reflect the light and increase the illumination. Different laser beams were used: 400 nm (10 mW Violet blue diode laser MDL-III-405; CNI Optoelectronics Tech., Changchun, China), 561 nm (MGL-III-561-5 mW; CNI Optoelectronics Tech.), 632.8 nm (10 mW Diode Laser 05-LHR-151; Melles Griot, Albuquerque, NM) and 730 nm (10 mW Red diode laser at 730 nm MLD-III-730; CNI Optoelectronics Tech.).

All the above-mentioned procedures and sperm handling were in the dark, except for brief periods of handling in which the spermatozoa were exposed to dim light. Control experiments of thermotaxis and sperm motility carried out with spermatozoa unexposed to light throughout the handling and experiment yielded similar results.

### Mouse sperm handling

Studies with mice were approved by the Institutional Animal Care and Use Committee of the Weizmann Institute of Science. The methods were carried out in accordance with the approved guidelines. Mice were sacrificed by cervical dislocation. Spermatozoa were collected from the cauda epididymis of 3-months-old C57BL/6 wild-type and C57BL/6 Rho^−/−^ knockout mice^56^, and suspended in modified human tubal fluid medium (HTF; MediCult) containing 1% BSA. Subsequently, the samples were incubated for 1 h under an atmosphere of 5% CO_2_ at 37 °C for capacitation. The sperm samples were handled with respect to light as described for human spermatozoa above.

### Thermotaxis assays

For thermotaxis experiments, the equally long two- or three-compartment thermoseparation tube having an internal diameter of 3.8 or 4.1 mm, respectively, was filled with spermatozoa in one of the compartments (#1 in Fig. 1a). The tube was placed in the thermoseparation device^8^, with the edge of the sperm-filled compartment at 34 °C and the other edge at 39 °C, creating a linear temperature gradient between these points, verified experimentally^7^. Following a 15 or 20 min separation period (for two or three compartments, respectively), the spermatozoa were collected from the warmer compartment and counted using a haemocytometer. For the no-gradient control, the tube was placed in a 34 °C water bath. The assays were performed blindly.

### Motility analyses

For motility analyses, subsequent to the treatments with the different inhibitors/conditions, spermatozoa were diluted to 2 × 10^6^ cells/ml (humans) in capacitating medium containing the same concentration of inhibitor used for the treatment, or 5 × 10^6^ cells/ml (mice) in HTF containing 1% BSA. The real-time analysis was carried out by homemade software as described^7^, except that the digitized data were collected from 4 × 15 s time segments (60 s in total) at 50 frames/s. The conditions for motion analysis followed the guidelines for CASA instruments^57^, excluding the recommended video framing rate for fast-swimming cells (60 frames/s).

### Examining the swimming response of human spermatozoa to temperature

Following capacitation, 3–7 × 10^5^ human spermatozoa/ml were divided to two 500 μl aliquots, one with added hydroxylamine (5 mM) and one without hydroxylamine. Samples from these aliquots were recorded under the microscope at 34 °C for 1 min, after which the temperature of the microscope stage was quickly shifted to 37 °C and the swimming behaviour of the cells was continued to be recorded for additional 1 min. The recordings were analysed for the motility parameters FD and percentage of hyperactivated spermatozoa (%HYP) as described^5^, employing a home-made computerized motion analysis system in MatLab.

### Examining the response of human spermatozoa to light

After capacitation, spermatozoa (3 × 10^6^ cells/ml) were recorded under a phase-contrast microscope for 2 or 5 min. During this time the light intensity was increased and decreased every 1 min by setting the microscope lamp to the lowest and highest light intensities intermittently, or the wavelength was changed by a specific filter from red (600 nm cutoff filter) to other wavelengths (>500 or <500 nm cutoff filters, and 450–550 nm interference filters) or to white light and back. The recordings were analysed as described^5^. Phototaxis was analysed by loading human spermatozoa (70 × 10^6^) in compartment #1 of two thermoseparation tubes (Fig. 1a), and placing the tubes in a box under a gradient of blue light (LED light from a flashlight through a 480 nm interference filter; 80 nm bandwidth). One tube was placed in a positive light gradient, i.e., stronger illumination at compartment #3, and another tube in a negative gradient, i.e., stronger illumination at compartment #1. A third tube, used as a negative control, was placed in the dark. Following 30 min incubation at 37 °C under these conditions, the number of cells in compartment #3 was counted.

### Detection of Ca^2+^_in_

Following incubation for capacitation, 10^7^ spermatozoa/ml were incubated with Fluo-3 (2.4 μM, Life technologies, Carlsbad, CA) for 30 min followed by two washes and final resuspension in Flushing medium to the same concentration. Prior to the fluorescence measurements, the relevant samples were incubated for 5 min with or without 1 or 10 μM NNC.

### Determination of the fraction of capacitated cells

The fraction of capacitated spermatozoa was calculated as the difference between the fraction of acrosome-reacted cells, measured with fluorescein isothiocyanate-*Pisum sativum* agglutinin, before and after stimulation with the Ca^2+^ ionophore A23187^58^.

### Detection of opsins’ mRNAs by quantitative Real–time PCR

Eyes and liver were recovered from six mice C57BL. Whole ejaculates of three human donors were pelleted and the seminal plasma removed. Total mRNA from each mouse organ and human ejaculate was extracted, followed by DNA removal using TRIzol^®^ Reagent and DNA-free^TM^ Kit (Life Technologies, Carlsbad, CA), respectively, according to the manufacturer’s instructions. Reverse transcription was performed using the SuperScript^®^ VILO^TM^ cDNA Synthesis Kit (Live Technologies, Carlsbad, CA) according to the manufacturer’s indications. For the detection of opsin mRNAs, specific primers flanking one intron to discriminate from possible undigested genomic DNA were designed (Supplementary Table S3 online). Real time–PCR was performed on a 7300 Real time PCR System (Applied Biosystems, Foster city, CA) using PerfeCTa^®^ SYBR^®^ Green FastMix^®^, ROX™ (Quanta Bioscience, Gaithersburg, MD). The relative levels of mRNA for each opsin were determined relative to a reference gene, glyceraldehyde 3-phosphatedehydrogenase, according to the ∆Ct method^59^.

### TIRF and confocal microscopy

After capacitation, human and mouse spermatozoa were washed three times in PBS by centrifugation (10,000 ×g for 1 min), the concentration adjusted to 2 × 10^6^ cells/ml, smeared on a microscope coverslip and air-dried. For TIRF, once dried, the coverslips were washed with H_2_O and observed in a TIRF microscope (AxioObserver Z1, Zeiss) with 100 × oil objective (NA = 1.45; 473 nm laser excitation, 535 ± 15 nm emission). Images were acquired with cooled electron-multiplying charge-coupled device camera (QuantEM512C; Photometrics, Tuscon, AZ). For confocal microscopy, following washing each coverslip with H_2_O, mounting medium (glycerol 90% in water) was added, the coverslip was sealed under another coverslip and then observed in a confocal microscope (Olympus IX81 FV1000, Tokyo; 406 nm excitation). Spectral images were acquired from the mid-plane of the cells (12 nm bandwidth and 10 nm step-size). The spectra of the head and midpiece were separately analysed with FV10–ASW 4.1 software (Olympus, Tokyo).

### Detection of retinal by liquid chromatography with tandem mass spectrometry

Chromato-graphic separation was done on a 50 × 2.1 mm internal diameter 1.7 μm UPLC BEH C18 column equipped with 5 × 2.1 mm internal diameter 1.7 μm UPLC BEH C18 pre-column (both Waters Acquity) with mobile phases A and B (5 and 95% acetonitrile in 0.1% formic acid, respectively; flow rate 0.5 ml/min; column temperature 30°C). The gradient was 0–4.5 min linear increase 30–50% B, followed by 4.5–5.0 min further increase till 100% B, 5.0–7.5 min hold at 100% B, 7.5–8.0 min back to 30% B, and 2 min equilibration at 30% B. For mass spectrometry, argon was used as the collision gas with 0.25 ml/min flow. The capillary voltage was 3.40 kV, source temperature 150°C, desolvation temperature 350°C, desolvation gas flow 640 l/min, cone voltage 15 V, source offset 20 V. Analytes were detected using multiple reaction monitoring with the following parameters: 328.3 → 236.4 (collision energy, CE = 10 eV) and 328.3 → 107.3 (CE = 15 eV) for retinal O-ethyl oximes, and 285.1 → 160.9 (CE = 10 eV) and 285.1 → 175.0 (CE = 16 eV) for retinals. The retention times observed for standards (both all *trans* and 11-*cis*) of retinal and retinal-oxime were 5.7 and 6.2 min, respectively.

### Western blots

After seminal fluid removal, 150 × 10^6^ cells/ml were diluted in 500 μl of SDS lysis buffer containing 6% SDS, 1 mM benzamide, 1 mM Na_3_VO_4_, 50 μM NaF, 50 μM pyrophosphate, 1 mM PMSF and 125 mM Tris-HCl pH7.5, supplemented with “cOmplete, EDTA-free Protease Inhibitor Cocktail” (Roche, Indianapolis, IN). The lysate was centrifuged (700  ×g; 5 min) and the supernatant collected for protein analysis. Proteins were resolved by SDS-PAGE (12% acrylamide) and transferred into a nitrocellulose membrane for immunoblotting following standard procedures.

### Immunocytochemistry

G_α t1_ was detected in immunocytochemical analysis as described^26^. For the detection of opsins, the following protocol was employed. In each experiment 20 × 10^6^ spermatozoa in an Eppendorf tube were fixed for 15 min with 500 μl paraformaldehyde (4% v/v in PBS) and washed three times with 1 ml PBS (3500 × g for 5 min). The final pellet was resuspended in 500 μl PBS containing BSA (0.5% w/v). Primary polyclonal antibodies (H-300 in the case of anti-rhodopsin antibody) were added at this point (diluted 1:100) and the samples were incubated overnight at 4 °C. The samples were then washed three times and resuspended in PBS containing 1% BSA, and then incubated at room temperature for 30 min with the secondary antibody Cy^TM^3-conjugated IgG goat anti-rabbit (1:40,000). After three washes in PBS as above the samples were mounted onto a slide and observed with a fluorescence microscope. The same protocol was followed for the immunostaining of rhodopsin in mouse spermatozoa, but the cells were not fixed with paraformaldehyde, and the primary antibody was RET–1. For double staining of rhodopsin and G_α t1_, spermatozoa were smeared onto a coverslip coated with poly-L-lysine, fixed with paraformaldehyde (4% v/v in PBS) for 5 min, washed three times with PBS for 5 min and permeabilized with Triton X-100 (0.1% v/v in PBS) for 5 min. After three 5 min-washes in PBS, the coverslips were incubated for 20 min in bovine fetal serum (10% v/v in PBS), followed by an overnight incubation with a mixture of the primary polyclonal antibodies [anti-rhodopsin I-17 and anti-Gα t1 K-20, each diluted 1:200 in bovine fetal serum (5% v/v in PBS)] at 4 °C. Following three washes in PBS, the coverslips were treated with TO-PRO^®^-3 (1 μM, Life technologies, Carlsbad, CA) to stain the nuclei. The coverslips were mounted onto a slide and images were taken with a fluorescence microscope.

### Measurement of cyclic nucleotide level

Capacitated human spermatozoa were distributed to aliquots (15 × 10^6^ cells in 600 μl). Some aliquots were incubated in a water bath at 34 °C for 15 min and then quickly moved to a 39 °C water bath. The other aliquots were first incubated in the 39 °C water bath for 15 min and then moved to the 34 °C bath. The samples used for obtaining the value prior to the shift (t = 0 in Table 3) were kept at the original temperature (34 or 39 °C) without shifting to the other temperature. All other samples were incubated for the time periods indicated in Table 3 after the temperature shift at t = 0, and then fixed by adding paraformaldehyde (1% v/v final concentration) for 5 min. Subsequently, the samples were washed three times with 1 ml PBS by centrifugation (13,000 × g for 1 min). For cAMP or cGMP extraction the final pellets were resuspended in 100 μl of HCl (0.1 M) and incubated for 15 min at room temperature. Following centrifugation (20,000 × g for 5 min), the supernatants were recovered and the cyclic nucleotide concentrations in them was determined by cAMP or cGMP Direct Immunoassay Kit (abcam^®^, Cambridge, UK) according to the manufacturer’s acetylation protocol.

## Additional Information

**How to cite this article**: Pérez-Cerezales, S. *et al.* Involvement of opsins in mammalian sperm thermotaxis. *Sci. Rep.* **5**, 16146; doi: 10.1038/srep16146 (2015).

## Supplementary Material

Supplementary Information

## Figures and Tables

**Table 1:** Motility parameters of human spermatozoa under various conditions.

Treatment (n)	VSL (μm/s)	VCL (μm/s)	VAP (μm/s)	LIN (%)	FD	MOT (%)
10 μM M119K (8)	76 ± 3*	95 ± 5*	78 ± 4*	81 ± 2*	1.27 ± 0.02	54 ± 7*
7 μM M119K (16)	**86 ± 4**	108 ± 5*	92 ± 4	80 ± 2*	1.19 ± 0.03	**66 ± 4**
3 μM M119K (9)	**82 ± 2**	116 ± 4	92 ± 3	71 ± 2	1.23 ± 0.02	**68 ± 3**
1 μM M119K (10)	**85 ± 3**	120 ± 4	96 ± 3	71 ± 2	1.24 ± 0.02	**63 ± 3**
Control (28)	**85 ± 3**	125 ± 3	99 ± 3	68 ± 2	1.31 ± 0.03	**67 ± 3**
NNC (21)	**67 ± 4**	101 ± 5*	75 ± 4*	66 ± 2	1.33 ± 0.03*	**68 ± 4**
Control (25)	**76 ± 2**	111 ± 3	87 ± 2	69 ± 2	1.27 ± 0.01	**70 ± 3**
SKF96365 (10)	**85 ± 8**	118 ± 8	96 ± 9	71 ± 5	1.24 ± 0.05	**77 ± 5**
Control (15)	**84 ± 4**	119 ± 5	96 ± 5	70 ± 2	1.26 ± 0.03	**66 ± 5**
Pyr3 (8)	**95 ± 4**	130 ± 4	107 ± 3	74 ± 3	1.27 ± 0.06	**73 ± 3**
Control (10)	**89 ± 3**	127 ± 5	101 ± 3	71 ± 4	1.31 ± 0.04	**69 ± 5**
NH_2_OH dark (12)	65 ± 3*	102 ± 5*	74 ± 5*	64 ± 2	1.20 ± 0.01*	**66 ± 5**
NH_2_OH light (10)	60 ± 4*	100 ± 6*	68 ± 5*	60 ± 2	1.19 ± 0.01*	**73 ± 4**
Light (10)	**80 ± 3**	123 ± 6	95 ± 5	65 ± 2	1.30 ± 0.03	**71 ± 4**
Control (18)	**78 ± 2**	123 ± 4	93 ± 3	64 ± 1	1.31 ± 0.03	**67 ± 4**
NH_2_OH dark (4)	58 ± 6*	91 ± 5*	64 ± 6*	63 ± 3	1.17 ± 0.01	**70 ± 8**
NH_2_OH 400 nm (5)	63 ± 5*	99 ± 4	71 ± 5	64 ± 4	1.22 ± 0.03	**66 ± 10**
400 nm (5)	**78 ± 3**	117 ± 2	89 ± 3	66 ± 2	1.25 ± 0.01	**74 ± 6**
Control (5)	**74 ± 4**	113 ± 5	87 ± 6	65 ± 1	1.29 ± 0.04	**71 ± 6**
NH_2_OH dark (9)	64 ± 4*	97 ± 4*	71 ± 4*	65 ± 2	1.19 ± 0.01*	**74 ± 3**
NH_2_OH 560 nm (9)	**66 ± 3**	102 ± 4*	74 ± 3*	65 ± 2	1.22 ± 0.02*	**65 ± 5**
560 nm (6)	**81 ± 4**	125 ± 6	96 ± 4	65 ± 2	1.28 ± 0.02	**68 ± 7**
Control (9)	**74 ± 2**	114 ± 5	86 ± 3	66 ± 2	1.32 ± 0.02	**64 ± 4**
NH_2_OH dark (5)	55 ± 5*	85 ± 6*	61 ± 6*	64 ± 4	1.18 ± 0.03	**68 ± 6**
NH_2_OH 630 nm (5)	**64 ± 5**	95 ± 5	70 ± 5	67 ± 4	1.18 ± 0.05	**75 ± 4**
630 nm (5)	**83 ± 4**	118 ± 5	94 ± 3	70 ± 3	1.27 ± 0.07	**65 ± 5**
Control (6)	**75 ± 4**	106 ± 4	84 ± 4	71 ± 4	1.29 ± 0.04	**71 ± 5**
NH_2_OH dark (5)	**64 ± 4**	94 ± 5	70 ± 4	67 ± 1	1.18 ± 0.04	**63 ± 4**
NH_2_OH 730 nm (5)	**65 ± 8**	99 ± 8	72 ± 9	65 ± 4	1.22 ± 0.02	**68 ± 9**
730 nm (5)	**76 ± 6**	115 ± 8	88 ± 7	66 ± 3	1.31 ± 0.03	**63 ± 7**
Control (5)	**75 ± 4**	110 ± 6	87 ± 5	68 ± 2	1.28 ± 0.05	**63 ± 2**
IBMX (9)	**62 ± 6**	115 ± 6*	77 ± 6	54 ± 4*	1.35 ± 0.05*	**61 ± 6**
Control (8)	**75 ± 4**	101 ± 3	82 ± 4	74 ± 3	1.19 ± 0.01	**63 ± 6**
Caffeine (8)	**85 ± 7**	134 ± 6	107 ± 7	63 ± 4*	1.28 ± 0.06	**60 ± 5**
Control (9)	**84 ± 5**	115 ± 8	97 ± 7	74 ± 4	1.24 ± 0.06	**50 ± 4**
Sildenafil (6)	**98 ± 8**	120 ± 6	108 ± 8	81 ± 3	1.13 ± 0.03	**67 ± 6**
Control (10)	**98 ± 5**	121 ± 5	108 ± 6	81 ± 2	1.11 ± 0.03	**64 ± 5**
IBMX (6)	**50 ± 5**	125 ± 12	59 ± 6	40 ± 3*	1.28 ± 0.04*	**64 ± 7**
2APB (6)	40 ± 4*	126 ± 17	49 ± 5*	32 ± 1*	1.30 ± 0.03*	**63 ± 8**
IBMX + 2APB (6)	32 ± 2*	139 ± 12*	44 ± 3*	24 ± 2*	1.6 ± 0.1*	**58 ± 9**
Control (6)	**57 ± 8**	110 ± 12	65 ± 9	51 ± 3	1.17 ± 0.01	**61 ± 8**

Human sperm samples were subjected to different drugs and treatments, detailed in the text, and then the motility was analysed (37 °C). Concentrations: 10 μM NNC, 1 μM SKF96365, 1 μM Pyr3, 5 mM hydroxylamine, 1mM IBMX, 2 mM caffeine, 0.1 μM sildenafil, 0.1 mM 2APB. Abbreviations: NH_2_OH, hydroxylamine; VSL, straight-line velocity (the time-average velocity of the sperm head along a straight line from its first position to its last position, expressed in μm/s); VCL, curvilinear velocity (time-averaged velocity of a sperm head along its actual curvilinear path, expressed in μm/s); VAP, average path velocity (velocity over an average path generated by a roaming average, expressed in μm/s), LIN, linearity [defined as (VSL/VCL)×100]^60^; FD, fractal dimension (a quantitative measure of the intensity of hyperactivation, expressing the degree to which the sperm trajectory fills a plane)^37^; MOT (%), percentage of motile spermatozoa. The values of VSL and MOT are in bold because the sperm accumulation in the warmer compartment in thermotaxis assays shown in Fig. 1a is expected to be linearly dependent on these parameters. The values are the mean ± SEM of the indicated n determinations (i.e., aliquots; 300–1800 cells for each aliquot). *P < 0.05 according to two-way ANOVA (relative to the non-treated control).

**Table 2:** Effect of hydroxylamine on temperature-dependent hyperactivation of human spermatozoa.

Treatment	T(°C)	FD	%HYP
No hydroxylamine	34	1.25 ± 0.01	13 ± 2
	37	1.17 ± 0.01*	4 ± 1*
With hydroxylamine	34	1.16 ± 0.01	2 ± 1
	37	1.15 ± 0.02	1.9 ± 0.5

The motility parameters FD and percentage of hyperactivated spermatozoa (%HYP) were consecutively analysed at 34 °C and 37 °C for 1 min periods under the illumination of a bright-field microscope in the presence and absence of hydroxylamine (5 mM). Each of the values shown is the mean ± SEM of 12 determinations. *P < 0.0001 according to two-way ANOVA for the difference from 34 °C.

**Table 3:** Temperature-stimulated changes in cyclic-nucleotide levels.

Time (s)	cGMP (pmol/10^7^ cells)		cAMP (pmol/10^7^ cells)	
	34 to 39°C	39 to 34°C	34 to 39°C	39 to 34°C
0	0.28 ± 0.06	0.28 ± 0.06	0.24 ± 0.06	0.9 ± 0.34
1	0.23 ± 0.06	0.36 ± 0.1	0.44 ± 0.12**	0.64 ± 0.25*
10	0.17 ± 0.02	0.31 ± 0.1	0.35 ± 0.13	0.62 ± 0.27
30	0.23 ± 0.03	0.2 ± 0.02	0.16 ± 0.06	0.87 ± 0.42

The values shown, obtained at the denoted time points after the indicated temperature shifts, are the mean ± SEM of 5–8 determinations. *P = 0.045; **P = 0.003 according to two-way ANOVA for the difference from time 0.
