# Supplementary material for: Involvement of opsins in mammalian sperm thermotaxis
Source: Sci Rep. 2015 Nov 5;5:16146. doi: 10.1038/srep16146 (PMC4633616; doi:10.1038/srep16146)
Supplement: Supplementary Information [file srep16146-s1.pdf]

Supplementary Information to: Involvement of opsins in mammalian sperm thermotaxis

Serafín Pérez-Cerezales, Sergey Borishpolets, Oshri Afanzar, Alexander Brandis, Reinat Nevo, Vladimir Kiss and Michael Eisenbach

Supplementary Discussion

Our findings suggest that opsins signal to the flagella via at least two signalling pathways, the PLC pathway and the cyclic-nucleotide pathway. A partial working model for these pathways, speculative at this stage, is shown in Fig. 6f.

The PLC pathway. The findings made in this study about the involvement of GPCRs and TRPC3 in sperm thermotaxis, taken together with our earlier findings[1] that the process also involves intracellular Ca^{2+}, PLC, and the IP_3R Ca^{2+} channel located on an internal Ca^{2+} store, suggest that the PLC pathway may involve the following steps. Temperature differences are sensed by opsins. The relevant opsin activates PLC via a G protein, resulting in the dissociation of the latter to $G_{\beta\gamma}$ and, presumably, $G_{\alpha q}$. Activated PLC catalyses the hydrolysis of PIP_2 to IP_3 and DAG. IP_3 binds to its receptor on an internal Ca^{2+} store and triggers Ca^{2+} release[2]. Simultaneously, DAG probably activates TRPC3 (and, perhaps, additional TRPCs) with a resultant Ca^{2+} influx[3,4]. The IP_3R- and TRPC3-dependent rise in Ca^{2+} affects flagellar bending[5] and, consequently, the swimming direction.

The cyclic-nucleotide pathway. The observations that transducin-1 (this study) and transducin-2[6] are present in human spermatozoa, that at least the former co-localizes with opsins, that PDE is involved in thermotaxis, and that temperature changes stimulate changes in the intracellular cAMP level suggest the following sequence of events in the cyclic-nucleotide pathway. Temperature-stimulated opsins modulate the PDE activity via the G protein transducin. The changes in PDE activity and, possibly, in adenylyl cyclase activity, transiently modulate the cAMP level in the cell, which indirectly modulates the concentration of intracellular Ca^{2+} and, consequently, flagellar bending, frequency of hyperactivation events[7,8], and swimming behaviour. It is not impossible that, as in sperm chemotaxis[9-13], changes in the level of cGMP, too fast to be detected by the method employed in this study, occur as well. For example, if the changes are similar to those in chemotaxis of sea urchin spermatozoa, it is possible that the changes detected by us at 1 s are the tail of the cAMP response, and that the level of cGMP did change but returned to the unstimulated level prior to our first measured point at 1 s. Resolving this question would require tools for measuring very rapid temperature-stimulated changes in the levels of cyclic nucleotides.

An intriguing question is why the reduction in thermotactic activity due to knocking out rhodopsin was 70%, a value larger than expected from the elimination of a single thermosensor out of several. We suggest that this implies the existence of a lattice-like array of thermosensors that is disrupted when rhodopsin is removed. Rhodopsin is known to have high supramolecular organization in the rod membrane in the retina[14] — organization that appears essential for the photosensitivity of rod cells[15]. The higher-than-expected reduction in sperm thermotaxis suggests that such supramolecular organization is also the case in mammalian spermatozoa. An array of highly organized opsins of different sorts and combinations may provide high sensitivity, as is the case in bacterial chemotaxis where an organized cluster of receptors provides high sensitivity and signal amplification[16].

Supplementary Figures

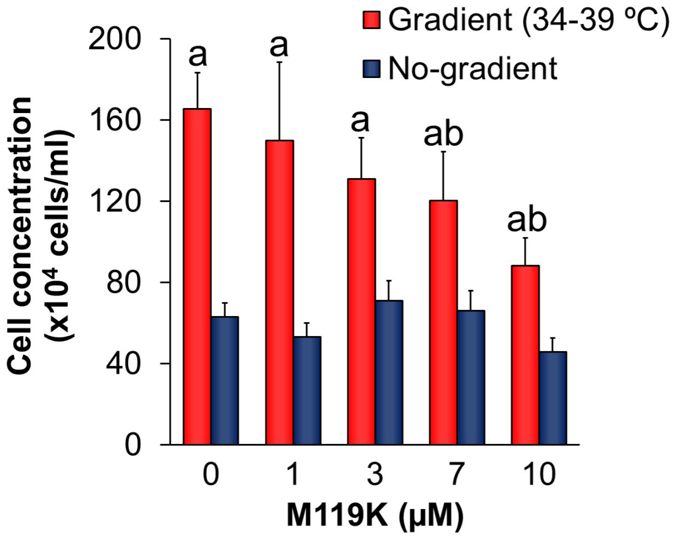

Figure S1. Effect of M119K on human sperm accumulation in two-compartment separation tubes. The columns stand for the accumulation of spermatozoa in the warmer compartment (or, in the case of no-gradient control, the compartment that did not contain spermatozoa at the onset of the experiment). The results are the mean±SEM of 15, 6, 5, 5, 9 repetitions for 0, 1, 3, 7, 10µM M119K, respectively. a, $P<0.05$ according to three-way ANOVA with respect to the no-gradient control at the same inhibitor concentration; b, $P<0.05$ according to three-way ANOVA with respect to the no-inhibitor control. There was no difference in the no-gradient accumulation (blue columns) between the presence and absence of the drug ($P=0.3$ according to three-way ANOVA).

References

1. Bahat, A. & Eisenbach, M. Human sperm thermotaxis is mediated by phospholipase C and inositol trisphosphate receptor Ca^{2+} channel. *Biol. Reprod.* **82,** 606–616 (2010).
2. Taylor, C. W. *et al.* Structural organization of signalling to and from IP_3 receptors. *Biochem. Soc. Trans.* **42,** 63–70 (2014).
3. Soboloff, J. *et al.* TRPC channels: integrators of multiple cellular signals. *Handbook Exp. Pharmacol.* **179,** 575–591 (2007).
4. Rohacs, T. Regulation of transient receptor potential channels by the phospholipase C pathway. *Adv Biol Regul* **53,** 341–355 (2013).
5. Lindemann, C. B. & Goltz, J. S. Calcium regulation of flagellar curvature and swimming pattern in Triton X-100--extracted rat sperm. *Cell Motil. Cytoskeleton* **10,** 420–431 (1988).
6. Spehr, M. *et al.* Particulate adenylate cyclase plays a key role in human sperm olfactory receptor-mediated chemotaxis. *J. Biol. Chem.* **279,** 40194–40203 (2004).
7. Armon, L. & Eisenbach, M. Behavioral mechanism during human sperm chemotaxis: Involvement of hyperactivation. *PLoS ONE* **6,** e28359 (2011).
8. Boryshpolets, S., Pérez-Cerezales, S. & Eisenbach, M. Behavioral mechanism of human sperm in thermotaxis — a role for hyperactivation. *Hum. Reprod.* **30,** 884–892 (2015).
9. Kaupp, U. B. *et al.* The signal flow and motor response controlling chemotaxis of sea urchin sperm. *Nature Cell Biol.* **5,** 109–117 (2003).
10. Teves, M. E. *et al.* Molecular mechanism for human sperm chemotaxis mediated by progesterone. *PLoS ONE* **4,** e8211 (2009).
11. Matsumoto, M. *et al.* A sperm-activating peptide controls a cGMP-signaling pathway in starfish sperm. *Dev. Biol.* **260,** 314–324 (2003).
12. Nishigaki, T. *et al.* A sea urchin egg jelly peptide induces a cGMP-mediated decrease in sperm intracellular Ca^{2+} before its increase. *Dev. Biol.* **272,** 376–388 (2004).
13. Yoshida, M. & Yoshida, K. Sperm chemotaxis and regulation of flagellar movement by Ca^{2}. *Mol. Hum. Reprod.* **17,** 457–465 (2011).
14. Fotiadis, D. *et al.* Atomic-force microscopy: Rhodopsin dimers in native disc membranes. *Nature* **421,** 127–128 (2003).
15. Dell'Orco, D. A physiological role for the supramolecular organization of rhodopsin and transducin in rod photoreceptors. *FEBS Lett.* **587,** 2060–2066 (2013).
16. Sourjik, V. & Berg, H. C. Receptor sensitivity in bacterial chemotaxis. *Proc. Natl. Acad. Sci. U.S.A.* **99,** 123–127 (2002).

Supplementary Tables

Opsin	Single staining (%)			Double staining (%)			Triple staining (%)
	EqR	PC	MP	EqR + PC	EqR + MP	PC + MP	
Rhodopsin	26	0	35	1	33	4	1
Encephalopsin	81	1	7	2	3	5	0
Melanopsin	0	97	1	0	0	2	0
Neuropsin	9	6	19	2	10	37	17
$G_{\alpha\, t1}$	1	4	0	4	4	34	53

Table S1. Distribution of opsins and $G_{\alpha\, t1}$ in human spermatozoa revealed by immunocytochemical analysis. The distribution of each opsin and $G_{\alpha\, t1}$ was determined in 300–600 spermatozoa by using the antibodies detailed in *Methods*. The grey background indicates >15% prevalence. Abbreviations: EqR, equatorial ring; PC, postnuclear cap; MP, midpiece.

Illumination	VSL (μm/s)	VCL (μm/s)	VAP (μm/s)	LIN (%)	FD	MOT (%)
Red(>600nm)	61 ± 2	109 ± 1	68 ± 2	56 ± 2	1.15 ± 0.01	63 ± 2
White (no filter)	61 ± 2	118 ± 1*	68 ± 2	51 ± 2*	1.18 ± 0.01*	61 ± 2
Red(>600nm)	59 ± 2	107 ± 1	66 ± 2	56 ± 2	1.15 ± 0.01	63 ± 3
Yellow(>500nm)	59 ± 2	113 ± 2*	66 ± 2	52 ± 1*	1.16 ± 0.01*	62 ± 3
Red(>600nm)	60 ± 2	105 ± 2	67 ± 2	57 ± 3	1.14 ± 0.01	65 ± 2
Blue(<500nm)	55 ± 4	96 ± 2	63 ± 3	58 ± 4	1.12 ± 0.01*	64 ± 3
Red(>600nm)	67 ± 7	110 ± 9	60 ± 8	56 ± 12	1.16 ± 0.02	61 ± 7
Green(450–550nm)	66 ± 10	108 ± 4	59 ± 11	55 ± 11	1.16 ± 0.02	56 ± 7
Low intensity	52 ± 1	107 ± 2	59 ± 1	49 ± 1	1.19 ± 0.01	56 ± 2
High intensity	53 ± 2	117 ± 3*	61 ± 2	46 ± 1*	1.20 ± 0.003	53 ± 2

Table S2. Effect of illumination on sperm motility. The experiment was carried out as described in Methods. The values shown are the mean±SEM of 8 determinations for the wavelength effects (*P<0.05 relative to the red filter according to three-way ANOVA) and 4 and 7 determinations for the effects of low- and high-light intensity, respectively (*P<0.05 relative to the low intensity according to three-way ANOVA).

Gene	Specie	Primer	Sequence 5'→3'	Amplicon length (bp)
GAPDH	Mouse	Forward	ACAACTTTGGCATTGTGGAA	133
		Reverse	GATGCAGGGATGATGTTCTG	
	Human	Forward	TGGTATCGTGGAAGGACTCA	132
		Reverse	CCAGTAGAGGCAGGGATGAT	
Blue Opsin	Mouse	Forward	TTCCCTCATCTGCTTCTCCT	110
		Reverse	TATGACTCACCTCCCGTTCA	
	Human	Forward	TGCTTCATTGTGCCTCTCTC	95
		Reverse	GTAGCTGACTCCTGCTGCTG	
Green/red Opsin	Mouse	Forward	GGCAATGTGAGATTTGATGC	107
		Reverse	CAGTACCTGCTCCAACCAAA	
	Human	Forward	CTACACCGTCTCCCTGTGTG	77
		Reverse	AGACCACCATCCATCTCTCC	
Rhodopsin	Mouse	Forward	GCTGTAATCTCGAGGGCTTC	144
		Reverse	CACCCATGATAGCGTGATTC	
	Human	Forward	ATGATGAACAAGCAGTTCCG	97
		Reverse	TCTTGGACACGGTAGCAGAG	
Encephalopsin	Mouse	Forward	CTGGTATCCCTGTTCGGAGT	139
		Reverse	GCACAGTGAGGGTGGTAATG	
	Human	Forward	TTTACCTTCGTGTCCTGCCT	124
		Reverse	CATAGGCCAGCACGGTTAG	
Melanopsin	Mouse	Forward	GTCCCAGACCATGCTCACTA	132
		Reverse	GAACATGTTTGCTGGTGTCC	
	Human	Forward	AGATCATGCTGCTGGTCATC	104
		Reverse	GTAGGGTGTCAGGACGTGTG	
Neuropsin	Mouse	Forward	TGGAAGCCTGATTACCATGA	89
		Reverse	GCTTTCTCTTCAGCCAGACC	
	Human	Forward	TTTCGACAGTCGGATCCATA	102
		Reverse	GCATAAGGAATCCAGGCAAT	

Table S3. Primers for the detection of opsins in mouse and human by Real-time PCR.
